# pCONUS for Distal Artery Protection During Complex Aneurysm Treatment by Endovascular Parent Vessel Occlusion—A Technical Nuance

**DOI:** 10.1007/s00062-020-00949-4

**Published:** 2020-09-07

**Authors:** Victoria Hellstern, Marta Aguilar-Pérez, Maike Dukiewicz, Gottlieb Maier, Hansjörg Bäzner, Hans Henkes

**Affiliations:** 1 Neuroradiologische Klinik, Kopf- und Neurozentrum, Klinikum Stuttgart, Stuttgart, Germany; 2 Neurochirurgische Klinik, Kopf- und Neurozentrum, Klinikum Stuttgart, Stuttgart, Germany; 3 Neurologische Klinik, Kopf- und Neurozentrum, Klinikum Stuttgart, Stuttgart, Germany; 4 Medizinische Fakultät, Universität Duisburg-Essen, Essen, Germany

## Introduction

The surgical occlusion of the parent arteries of intracranial aneurysms can be accomplished by either proximal clipping [1] or obstructing the distal outflow [2]. Endovascular parent vessel occlusion (PVO) was introduced in the 1980s [3]. In ruptured aneurysms, parent artery reconstruction might be associated with a better outcome [4]. For PVO to be a feasible option, a sufficient collateral supply is required. Vessel reconstruction, however, requires suitable proximal and distal landing zones for the stent or flow diverter. Difficulty in accessing the distal vessel(s), posthemorrhagic vasospasm or major vessel diameter discrepancies between the proximal and distal arteries may render stenting impossible. In fusiform and dissecting aneurysms, the distal vessel(s) can be incorporated into the aneurysm. These distal vessels can, however, be part of the collateral circulation downstream to the aneurysm. If the parent vessel and the aneurysm are occluded with coils the risk of ischemic complications must be considered. The coils will follow the contours of the aneurysm dome, thus possibly occluding the integrated distal vessel(s). A pCONUS (phenox, Bochum, Germany) deployed underneath the aneurysm dome in front of the distal vessels helps to prevent occluding the arteries. The coils are then placed proximal to the pCONUS petals.

## Illustrative Case #1

A comatose 20-year-old female was brought to the emergency room with a massive subarachnoid hemorrhage (SAH). Despite the poor prognosis, the decision taken by the surgical and interventional neurovascular team was to attempt endovascular treatment. This decision was justified by the young age, undilated pupils, normal angiographic circulation time and two hemorrhages within a matter of hours. Cerebral digital subtraction angiography (DSA) showed suspected dissecting aneurysms in the cavernous segment of the left internal carotid artery (ICA) (22 mm), of the left distal V4 segment (8.5 mm diameter, 14 mm length) and of the basilar artery (BA) trunk (18 mm diameter, 25 mm length). Based on size, location and shape, the BA aneurysm was considered the most likely source of the SAH since the hemorrhage pattern in cranial computed tomography (CT) did not provide further information for the identification of the ruptured aneurysm. A rotational DSA with 3D reconstruction with contrast medium injected into the left vertebral artery (VA) confirmed that both posterior cerebral arteries (PCAs) and both superior cerebellar arteries (SCAs) were integrated into the aneurysm dome. Injecting contrast medium into both ICAs during a balloon occlusion test (BTO) of the vertebral artery junction with a 4/15 mm Scepter C balloon (MicroVention, Aliso Viejo, CA, USA) showed a small caliber left posterior communicating artery (PcomA); however, there was no PcomA on the right. The PVO of the BA appeared to be the only viable treatment option, since the caliber discrepancy between the PCAs (as the only possible distal landing zone) and the proximal BA rendered both stent-assisted coiling and flow diversion technically unfeasible.

**Fig. 1:**
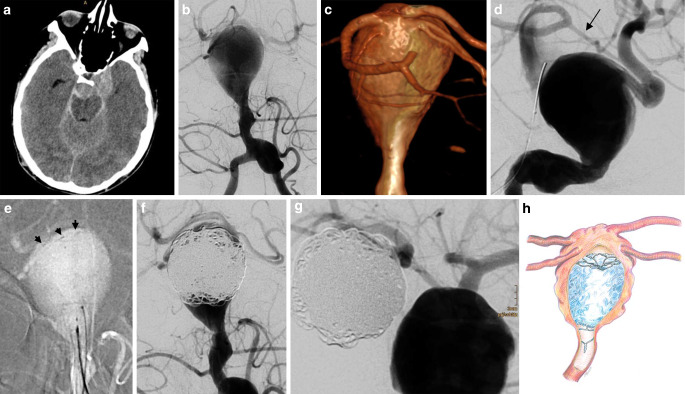
Endovascular parent vessel occlusion of a ruptured dissecting basilar artery aneurysm. Axial CT without contrast (**a**) showed an acute SAH, most prominently in the basal cisterns, as well as moderate hydrocephalus with dilatation of the temporal horns of the lateral ventricles. 2D DSA (**b**) and 3D DSA (posterior view (**c**)) showed the dissecting BA trunk aneurysm. The origins of both the SCA and the PCA origins were incorporated into the aneurysm dome. BTO of the VA junction showed the spontaneously not opacified left PcomA (*arrow*) (**d**). A pCONUS2 with a 12 mm wingspan was deployed adjacent to the SCA and PCA origins (*arrowheads*) (**e**) and the aneurysm proximal to the pCONUS2 was occluded with coils (**f**). Injecting contrast medium into the VA right after coil insertion while thrombus between the coil loops had still not completely formed, showed the patency of the SCAs and PCAs (**f**). At this point, the left PcomA had already been recruited (**g**). Illustration of the position of the pCONUS2 and the coils (**h**). The patient eventually died as a consequence of the SAH (artwork by Mark Hobert)

At the onset of the procedure, 2 × 500 mg Aspirine (ASA) intravenously (IV) was administered. Via 5F guiding catheters in both VAs, a pCONUS2 HPC with a 12 mm petal wingspan was deployed into the aneurysm dome adjacent to the origin of the PCAs and SCAs. The pCONUS2 was held in place while the aneurysm sac was densely packed with 43 detachable coils. A subsequent contrast injection into the left ICA at a pharmacologically elevated blood pressure of 160/90 mmHg confirmed the patency of the left PcomA, PCAs and SCAs. Approximately 1.5 h after the BA aneurysm had been occluded, both pupils were dilated and distorted. A drain was inserted into the enlarged right lateral ventricle. The intracranial pressure measured 19 mmHg. Cranial CT 17 h after the second (preprocedural) SAH showed massive infarcts of both cerebral hemispheres with downward herniation. Intensive care was withdrawn and the patient died 1 day after the SAH (Fig. 1).

## Illustrative Case #2

A 21-year-old woman presented at the referring hospital having had difficulty finding words for 3 days, accompanied by hypoesthesia of the right leg and the IVth and Vth fingers of the right hand. A magnetic resonance imaging (MRI) showed scattered lesions with diffusion restriction in the left middle cerebral artery supply territory, a partially thrombosed intradural fusiform aneurysm of the left ICA (31 mm diameter including the thrombus, 7.5 mm diameter of the perfused part, 33 mm length). A sulcal SAH was visible on FLAIR images over the left hemisphere. It was decided to attempt a reconstruction of the dissected left distal ICA through flow diversion. Under confirmed P2Y12 platelet receptor inhibition with prasugrel, four HPC coated flow diverter stents were implanted, starting just distal to the ophthalmic artery origin. Coverage of the entire fusiform aneurysm was, however, not possible. The diameter discrepancy between the M1 segment as the distal landing zone and the fusiform aneurysm itself, alongside a severe, mechanically induced vasospasm of the MCA, prevented proper implantation of a distal flow diverter. The procedure was unsuccessful but well tolerated.

The PVO of the left ICA was carried out 5 days later. With the patient under general anesthesia, BTO of the left ICA was started. With the left ICA occluded with a 4/30 mm Transform C balloon catheter (Stryker Neurovascular, Fremont, CA, USA), injecting contrast medium into the right ICA and the left VA showed:

- the left A1/M1 junction distal to the aneurysm, not integrated into the vessel dilatation,
- the left PcomA/left ICA junction integrated into the aneurysm,
- the left AchoA originating from the aneurysm.

These circumstances required the aneurysm to be occluded while still preserving the most distal part of the dilated vessel. A pCONUS1 with a 4 mm shaft diameter, 20 mm shaft length and 5 mm petal wingspan was implanted into the flow diverters as a scaffold for a second pCONUS. A pCONUS2 with a 15 mm petal wingspan was deployed inside the distal part of the aneurysm, just proximal to the origin of the anterior choroidal artery (AchoA), with the petals creating a protective fence for the PcomA and the AchoA. The vessel lumen proximal to the pCONUS petals was densely packed with 28 coils. The patient’s blood pressure was pharmacologically elevated to a target value of 160 mmHg for 3 days with a mean arterial pressure (MAP) of >100 mmHg. Antiaggregation was switched from prasugrel to 2 × 100 mg ASA per os (PO) daily. After the procedure, hemiparesis of the right-hand side temporarily persisted without a correlate on cranial CT, resolving within 1 day. A MRI 6 days after the PVO showed small diffusion restricted lesions of the posterior limb of the left internal capsule and of the left mesial temporal lobe. A DSA 1 week and 7 months after the PVO confirmed the collateral supply of the left anterior circulation via the AcomA and PcomA with a patent left-hand AchoA. The patient was discharged home 11 days after the left ICA PVO in a clinical condition rated according to the National Institutes of Health stroke scale (NIHSS) 1 and was asymptomatic at 7‑month follow-up (Fig. 2).

**Fig. 2:**
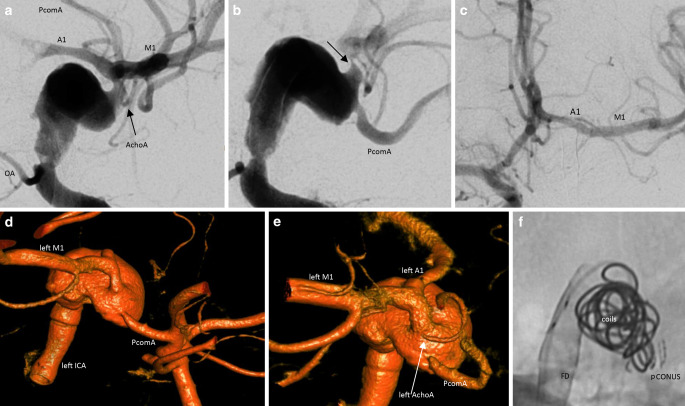
Endovascular parent vessel occlusion of a dissecting distal ICA aneurysm. 2D DSA (**a**) showed a fusiform aneurysm of the supraclinoid segment of the left ICA with the dissection just distal to the ophthalmic artery (*OA*) (*arrow*: anterior choroidal artery). This left anterior oblique (LAO) projection also demonstrates the distal end of the aneurysm and the relation to the A1 and M1 segments. After the implantation of four flow diverters, the left M1 segment was catheterized. It was, however, not possible to gain catheter access to the left M1 segment due to severe mechanically induced vasospasm (*arrow*), (**b**). Contrast injection into the right ICA showed that this vasospasm had not affected the left A1/M1 connection (**c**). BTO of the left ICA with injection of the left VA showed that the PcomA/ICA connection was integrated into the aneurysm (**d**). The origin of the left AchoA (*arrow*) from the aneurysm was visualized by injecting contrast into the right ICA during BTO of the left ICA (**e**). A pCONUS construct inside the fusiform aneurysm was used to prevent the inserted coils from occluding the aneurysm dome (**f**)

**Fig. 2:**
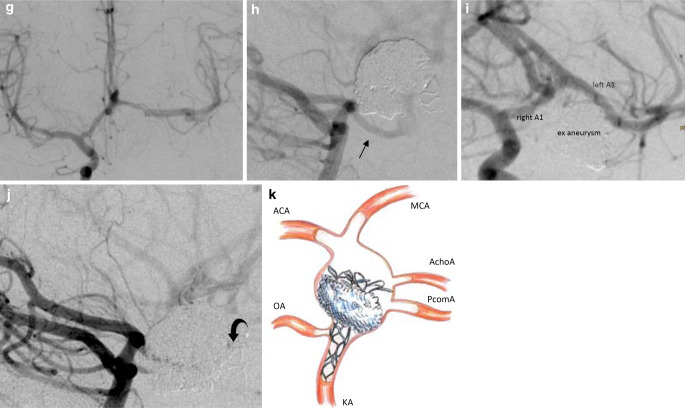
(continued). DSA after the left ICA PVO showed cross flow via the AcomA (**g**) and significant supply via the left PcomA (*arrow*) (**h**). A 7‑month DSA follow-up confirmed the patency of collateral vessels (**i**, **j**). Illustration showing the inverted pCONUS-assisted PVO with the petals protecting the distal arteries (**k**) (artwork by Mark Hobert)

## Discussion

The treatment of giant aneurysms (diameter >25 mm) may result in an immediate neurological deterioration in about 50% of cases, with PVO being associated with a good outcome and SAH with a poor outcome [5]. The PVO is considered a bail out strategy for treating intracranial aneurysms. Obliterating such a vessel and its aneurysm can be achieved by clip ligation, coil occlusion or with liquid embolic agents. In order to avoid hemodynamic ischemic damage to the dependent brain parenchyma, it is imperative to perform angiographic visualization and possibly functional testing of the collateral circulation for the target vessel beforehand. [6]. If the natural collaterals are insufficient, a surgical bypass from the external carotid artery to the middle cerebral artery (MCA) is a viable option [7]. In carefully selected patients, very low complication rates of PVO have been achieved [6]. In a series of 274 patients with unruptured aneurysms and elective treatment with PVO, Nishi et al. [8] encountered a 20% complication rate with 5.8% morbidity and 0.7% mortality. In a meta-analysis, Cagnazzo et al. [4] compared the results of reconstructive and deconstructive endovascular strategies for very large and giant intracranial aneurysms in 894 published cases. A PVO yielded the best results for unruptured aneurysms in the anterior circulation. For PVO performed on ruptured aneurysms in the acute phase, a complication rate of over 50% was reported [9]. For both ruptured and unruptured ICA aneurysms, ischemic stroke related to the AchoA and more distal perforators is a known issue following PVO [10].

Coil occlusion per se is meant to completely occlude the aneurysm fundus. Preserving a specific segment of the aneurysm circumference can be difficult, if not impossible to achieve with coils alone. The original purpose of the pCONUS was to retain coils inside wide-necked bifurcation aneurysms, preventing them from occluding the parent vessel adjacent to the aneurysm neck [11]. The usage described is the reverse situation. The pCONUS is implanted inside the aneurysm fundus with the device’s petals protecting the patency of the distal arteries, which enables dense coil occlusion proximal to the crown and around the stent shaft; however, it is important to prevent this ancillary device from dislodging during the movement inherent in coil insertion. To this end, it is recommended that the device remains attached to its insertion wire until the coil occlusion has been completed. Distal thrombus migration during coiling is also a concern; therefore, our patients were under platelet inhibition and moderate anticoagulation.

## Abbreviations

ACA: Anterior cerebral artery
AchoA: Anterior choroidal artery
BA: Basilar artery
BTO: Balloon test occlusion
DSA: Digital subtraction angiography
ICA: Internal carotid artery
MCA: Middle cerebral artery
PCA: Posterior cerebral artery
PcomA: Posterior communicating artery
PVO: Parent vessel occlusion
SAH: Subarachnoid hemorrhage
SCA: Superior cerebellar artery
VA: Vertebral artery
